# Photobiomodulation Therapy for the Symptoms Related to Temporomandibular Joint Disk Displacement

**DOI:** 10.1155/2023/5947168

**Published:** 2023-04-13

**Authors:** Piotr A. Regulski, Kazimierz T. Szopinski, Špela Levičnik-Höfferle

**Affiliations:** ^1^Department of Dental and Maxillofacial Radiology, Faculty of Medicine and Dentistry, Medical University of Warsaw, Binieckiego 6 St, Warsaw, Poland; ^2^Piotr Regulski Dental Office, Grochowska 278 Street, Warsaw, Poland; ^3^Gamma Medical Center, Broniewskiego 3, Warsaw, Poland; ^4^Fotona d.o.o., Stegne 7, Ljubljana SI-1000, Slovenia

## Abstract

Pain related to temporomandibular disorders (TMD) is a common problem that can significantly influence a patient's quality of life. Laser photobiomodulation (PBM) has been reported as a promising method in medicine for wound and bone healing, pain relief, and treatment of the temporomandibular joint (TMJ). Our clinical case aimed to demonstrate the effectivity of PBM using 1064 nm Nd:YAG laser for the treatment of pain and restricted mandible movement in a patient with anterior disk displacement of the left TMJ, using subjective (pain on visual analogue scale - VAS) and objective outcome measures [dynamic magnetic resonance imaging (MRI)]. PBM was performed on the left condyle in four sessions using a 1064 nm Nd:YAG laser with a flat-top handpiece. Results after 10 weeks showed an increase in mouth opening and a painless joint on palpation, with no reported adverse effects. An MRI of the TMJ confirmed the left disk displacement, however, with no signs of inflammation or effusion and with less pronounced disk deformity as compared with the first MRI examination. Use of PBM with Nd:YAG laser may be an efficient method for the management of orofacial pain in patients with acute and chronic TMJ disk displacements and may reduce the recovery time.

## 1. Introduction

Temporomandibular disorder (TMD) is a condition affecting jaw movement. It is caused by various clinical problems associated with the temporomandibular joint (TMJ), myofascial muscles, and other structures [1], which can significantly influence a patient's quality of life [2]. The main symptoms of TMD involve non-dental orofacial TMJ pain and clicking, pain in the oral or myofascial masticatory muscles, and abnormal jaw movement [3, 4]. Pain related to TMD is a common problem in modern society [4] and may be challenging due to complex pathophysiology and associated psychosocial co-morbidities [5].

Treatments for TMD range from physiotherapy, manual therapy, occlusal splint, laser therapy, oral and injectable pharmacotherapy, and even surgery in more severe cases. Treatments with occlusal splints, exercises, and massage therapy are often successful and are the most commonly reported methods and usually the first choice for treatment of pain related to TMD [6–8]; however, severe acute or chronic pain due to inflammation or degeneration may call for additional treatment methods to help restore mandibular function and offer relief from associated symptoms [9]. Many of the therapies are not able to provide long-term results [10].

Laser therapy, specifically photobiomodulation (PBM) using different laser wavelengths, has been used in various fields of medicine for the reduction of pain, modulation of inflammation, and acceleration of healing [9, 11–13]. PBM was proven to modulate inflammatory and anti-inflammatory cytokines, stimulate osteoblasts, enhance perfusion to joint structures, and reduce nerve stimulation [14–16]. Laser light absorbed by tissue can also stimulate the lymphatic system and improve microcirculation [17]. The ability of PBM to reduce inflammation and decrease oxidative stress is also beneficial in cases of muscle injury and fatigue [18]. Studies suggest that PBM with red or near infra-red laser results in substantial effects in patients with TMD, improving chronic pain, functionality, and quality of life [19].

Previous studies on the application of 1064 Nd:YAG laser showed that it was as effective as occlusal splints for pain reduction in patients with myofascial pain dysfunction syndrome [20]. Both the Nd:YAG and 810 nm diode lasers were effective for the treatment of subjective tinnitus related to TMDs [21]. Moreover, successful cases of wound healing, pain relief, and bone healing [22] as well as treatment of TMJ and other orofacial pain [23, 24] have previously been reported also using 1064 nm Nd:YAG laser with a larger 1 cm^2^ flat-top Genova® handpiece (Fotona d.o.o., Slovenia) [25]. Results showed immediate pain reduction and an increase in mouth opening, which additionally improved with each subsequent treatment for patients with TMJ caused by malocclusion [24]. Larger spot-size handpieces with a flat-top beam profile are especially useful as they enable fast and reproducible treatments and deeper penetration of the laser light to reach the desired structures [26–28].

Our clinical case aimed to demonstrate the effectivity of PBM using 1064 nm Nd:YAG with handpieces that provide a flat-top beam profile (Genova®) for the treatment of pain and limited mouth opening mobility in patients with anterior disk displacement of the left TMJ using subjective [pain on visual analogue scale (VAS)] and objective outcome measures [magnetic resonance imaging (MRI) of TMJ].

## 2. Case Presentation

**Figure 1:**
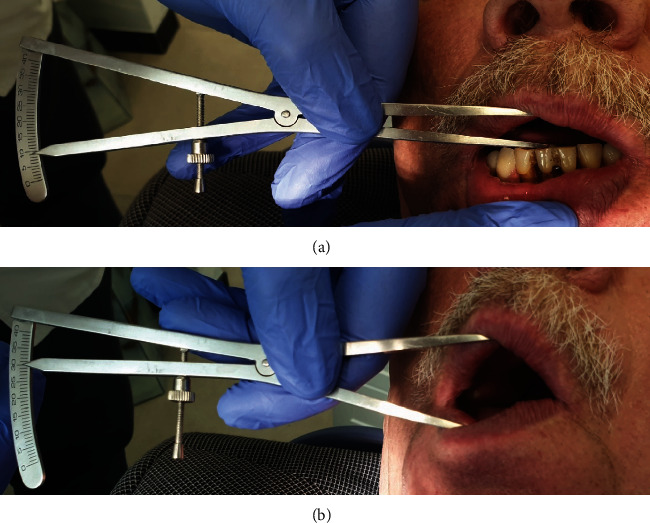
(a) Clinical examination of patient on initial visit revealed limited and painful mouth opening to 9.5 mm. (b) Examination at 10-week follow-up after the treatment revealed increased mouth opening to 30 mm.

A 69-year-old male presented with complaints of pain in the area of the left TMJ and limited mouth-opening mobility for 2 months. On physical examination, the patient had restricted mandible movements to the right side and severe pain in the left TMJ on palpation. The patient subjectively rated the pain at 75 on the VAS, of which 100 is the maximum pain that the patient could imagine [29]. The pain was aggravated during chewing. At the same time, the patient noted a discontinuation of an audible clicking sound previously present. Physical examination revealed a limited and painful mouth opening to 9.5 mm (Figure 1) with a shift of the mandibular midline to the left at opening. The patient had restricted mandible movements to the right and severe pain on palpation posteriorly or laterally to the left TMJ. Other joints and muscles were symptom-free.

**Figure 2:**
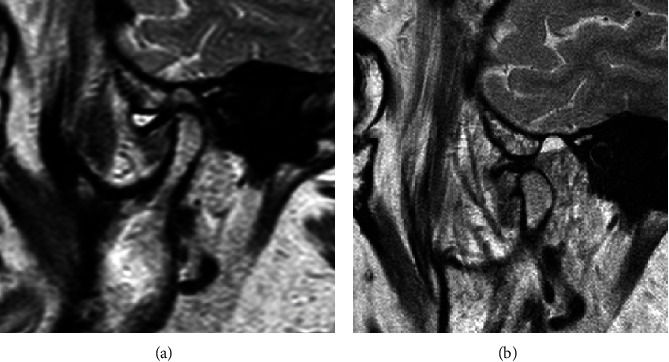
Initial MRI of the left TMJ with the patient's mouth closed (a) and open (b).

**Figure 3:**
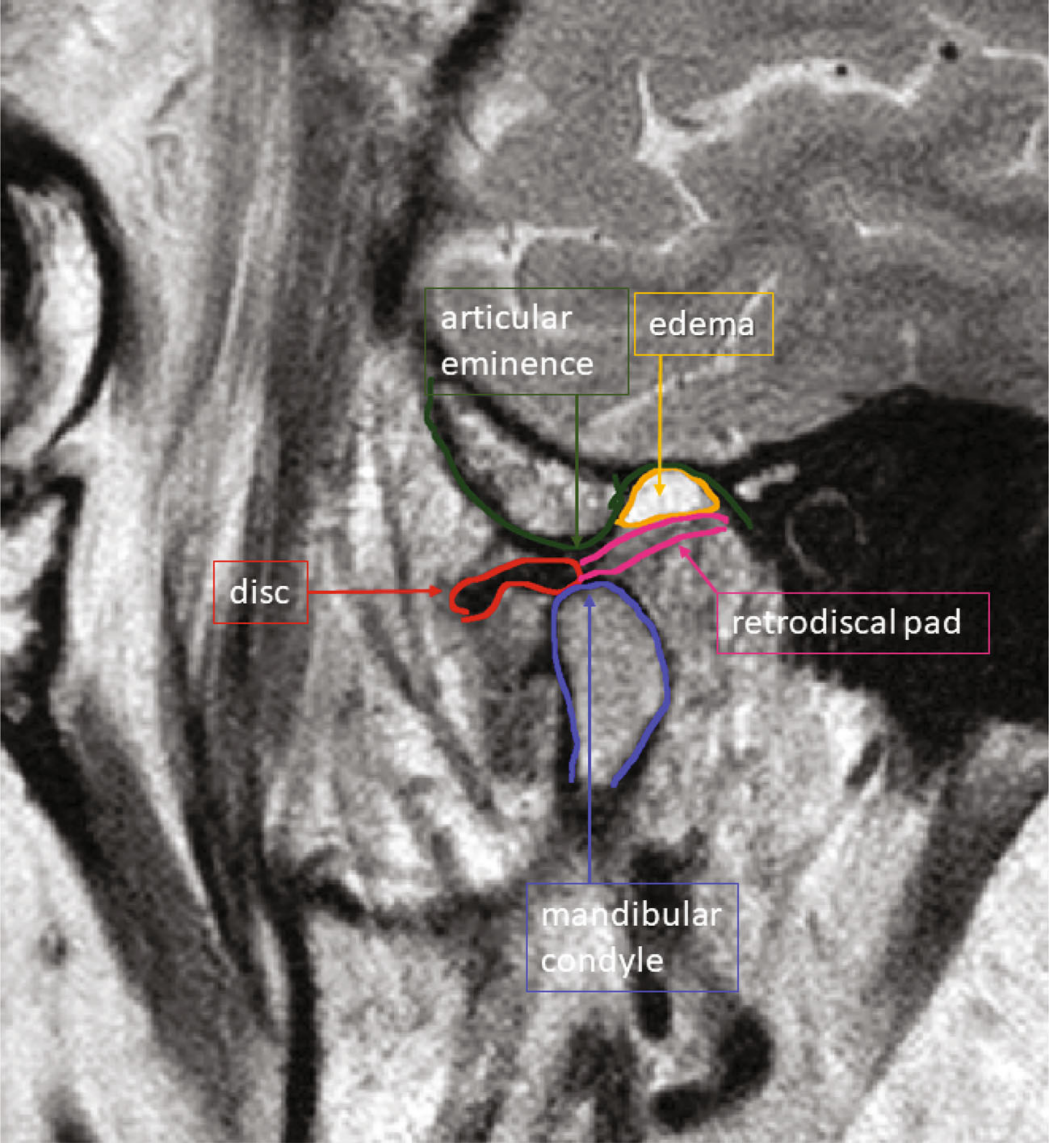
Initial MRI of the left TMJ with the patient's mouth open revealed anterior disk displacement of the left articular disk with significant anatomical deformity (thickening of the posterior edge) and effusion in the left TMJ. The dynamic sequences revealed limited condyle movement, disk deformity, and displacement without reduction on opening. Joint effusion was present.

The patient was referred for a dynamic MRI of the TMJs, which was performed using a 1.5-T MRI unit (Philips Achieva) with an eight-channel head coil (8-channel SENSE Head coil). The proton density (PD) spin echo sequence and T2-weighted turbo spin echo sequence were obtained for the TMJ in sagittal oblique and coronal oblique projections. The MRI revealed anterior disk displacement of the left articular disk with significant anatomical deformity (thickening of posterior edge) and effusion in the left TMJ. The right TMJ was symptomless. To illustrate the limitation with mouth opening, the MRI images of the left TMJ were obtained with the patient's mouth closed (Figure 2) and open (Figures 2 and 3), and in the intermediate positions stabilized with a specially designed bite block [30]. The dynamic sequences revealed limited condyle movement, disk deformity, and displacement without reduction on opening. Joint effusion was present.

The study conforms to the recognized standards (Helsinki Declaration) and was approved by the local ethics committee (Bioethics Committee's reference number: AKBE/85/2021). The patient's written consent to publish his information has been obtained before submission to the journal.

**Figure 4:**
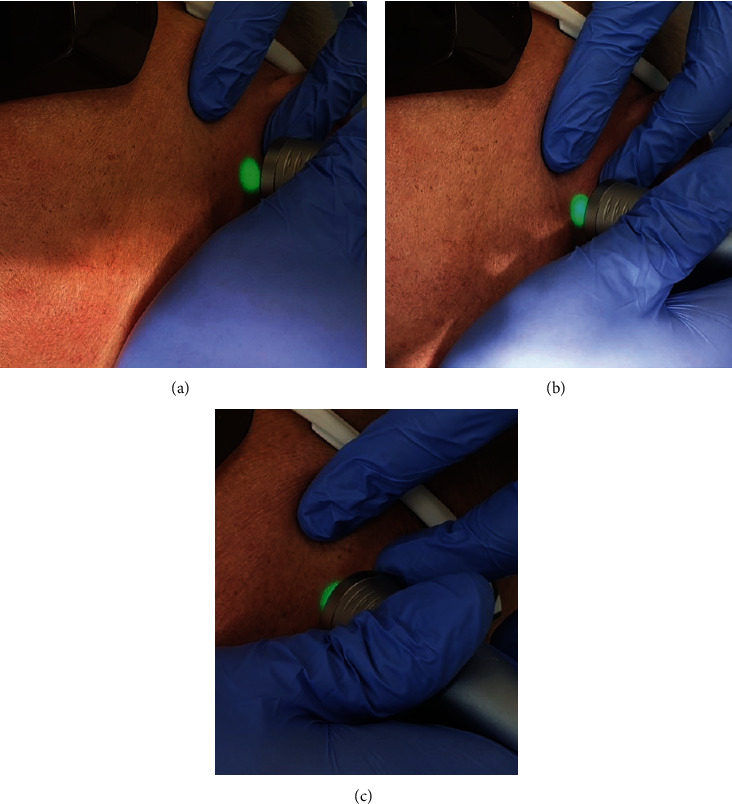
Photobiomodulation using LightWalker laser (Fotona d.o.o.) was performed with a flat-top Nd:YAG handpiece. The laser was applied in three places: posteriorly (a), above (b), and anteriorly (c) to the condyle. Four treatment sessions were performed.

PBM using a LightWalker® laser (Fotona d.o.o.) was performed with an Nd:YAG Genova® handpiece with 1 cm^2^ spot size according to the standard protocol [0.5 W/cm^2^ irradiance, 10 Hz repetition rate, 100 *μ*s pulse duration (Micro Short Pulse - MSP pulse), and 60-second irradiation on each spot in stamping technique, resulting in 29 J/cm^2^/minute]. The laser was applied in three places: anteriorly, above, and posteriorly to the condyle (Figure 4). The procedure was performed four times. The first three sessions were performed consecutively every other day, whereas the fourth session took place after a 3-week delay. A soft diet was recommended. No non-steroidal anti-inflammatory drugs were prescribed.

**Figure 5:**
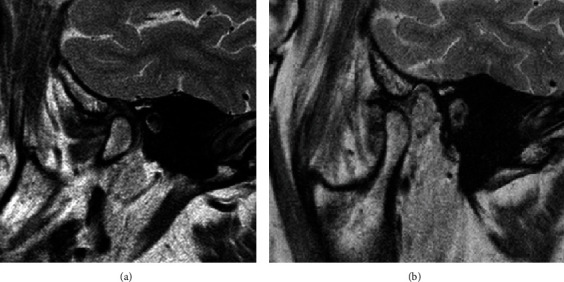
Dynamic MRI of the TMJ at 10-week follow-up with patient's mouth closed (a) and open (b).

**Figure 6:**
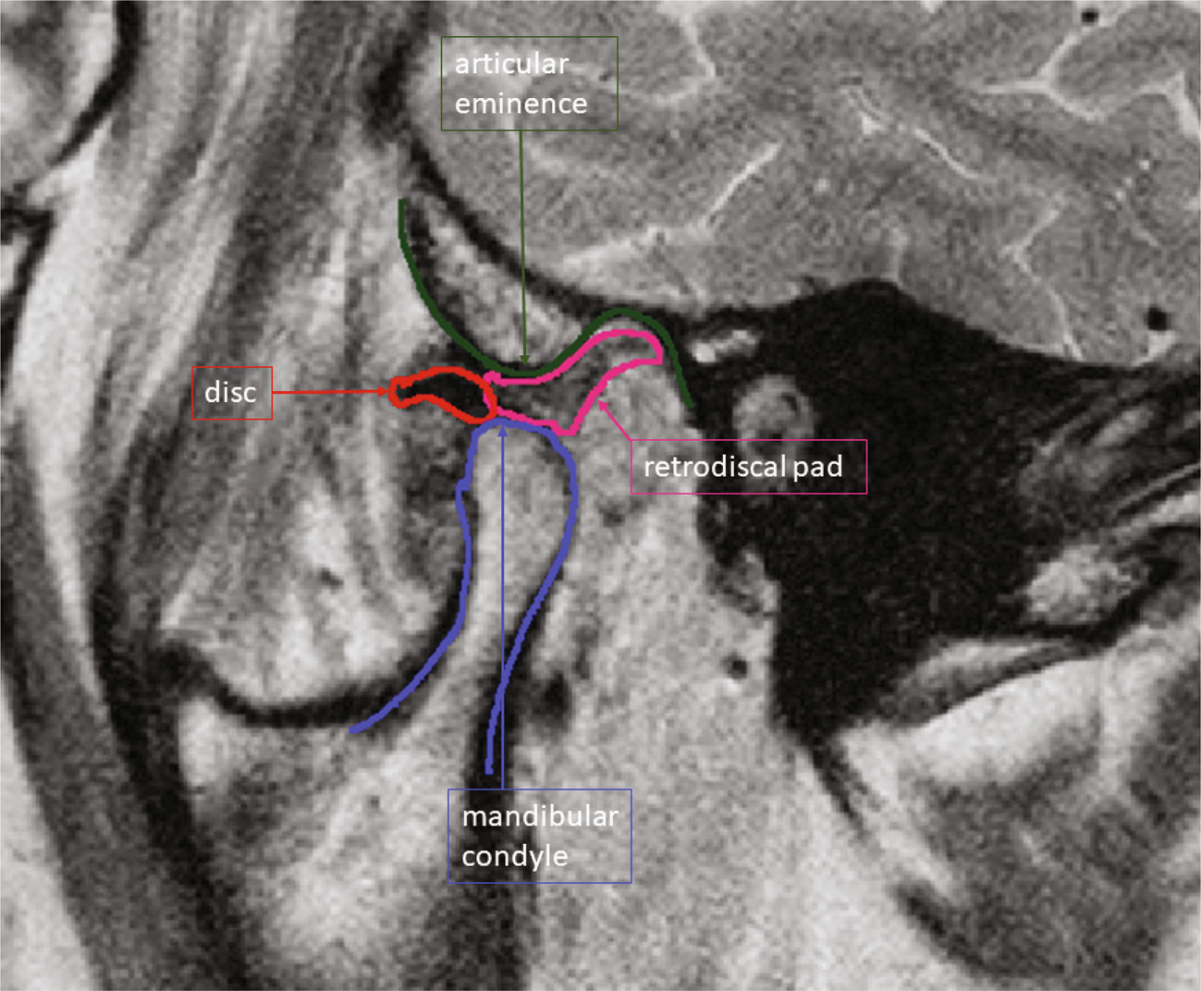
Dynamic MRI of the TMJ at 10-week follow-up with patient's mouth in the open position revealed persistent left disk displacement, however, without signs of inflammation or effusion. The condyle moved further forward than in the first MRI examination and reached the top of the articular eminence.

The pain reduced immediately after treatment. The next day, the pain increased again temporarily. After three weeks, the follow-up revealed increased mouth-opening mobility up to 15 mm with accompanying pain reduction. The next follow-up was done after 10 weeks from the initial visit. The patient was referred for a dynamic MRI of the TMJ. PD sagittal sequences with the patient's mouth closed (Figure 5) and in intermediate and open positions (Figures 5 and 6) were performed. No radiologic abnormality of the right TMJ was demonstrated. The study confirmed persistent left disk displacement, however, without signs of inflammation or effusion. The demonstrated disk deformity was less pronounced than that in the first MRI examination. In the maximum opening position, the condyle moved further forward than in the first MRI examination and reached the top of the articular eminence. Clinical examination revealed additional mouth opening mobility to 30 mm (Figure 1), and the joint was painless on palpation. The patient subjectively rated the ailments at 10 on the 0–100 VAS scale. No adverse effects were reported.

## 3. Discussion

The presented patient was diagnosed with TMJ disk displacement without reduction. Non-reducing disk displacement is a result of progression of reducing displacement and can be divided into an acute and a chronic phase. The acute phase is characterized by an inability to open the mouth more than 20 mm along with mandibular deflection to the affected side, caused by a limitation of condylar translation by the displaced, blocking disk, and by the contraction of mastication muscles. Tenderness at palpation of the affected joint is usually reported by the patient. The laterotrusive movements are painful on the affected side, and the movement is limited on the unaffected side. Patients usually report dull pain, which starts when previously observed joint clicking discontinues [4].

The acute phase of non-reduced disk displacement progresses into the chronic phase, which is characterized by a significant reduction in pain and gradual improvement of mandibular movements with a reduction of mandibular deflection. In addition, effusion, which is a feature of acute inflammation, is not visible on MRI examination. According to the available literature, the time required for this progression depends upon the method of treatment, comorbidities, and the patient's individual characteristics. The treatment success is considered the transition from the acute phase into the chronic phase with significant pain reduction. Alternative treatment options for TMJ might include splint therapy and occlusal treatment, physical therapy, medications, and soft diet [31]. If conservative treatment methods are unsuccessful, surgical intervention might be required [32, 33]. Sano and Westesson showed that 18-month splint therapy resulted in a 55% success rate in pain reduction; however, a similar success rate was obtained for untreated patients [7]. Linde et al. presented a method of transcutaneous electric nerve stimulation with a success rate of 6% [6]. Kai et al. showed a 75% pain relief success in patients who underwent 2-year occlusal splint therapy and subsequent additional occlusal treatment [8]. The invasive treatment options include arthroscopy, which involves making a small incision and using a camera to see the inside of the joint, and open-joint surgery, in which a larger incision is made, and the joint is accessed directly. However, surgery is usually reserved for severe cases that do not respond to other treatments [32].

The pain localized in the TMJ region not only might be associated with temporomandibular pathologies but also should be differentiated with other diseases. The lacerating pain is associated mainly with primary neuralgias and throbbing pain with vascular conditions. The dull pain originates primarily from muscle contraction and aching pain from inflammatory conditions. The sharp, burning pain can be related to nerve entrapment associated with inflammatory, benign, or malignant origin [4]. Therefore, in this case, MRI was performed to exclude other diseases.

This case report presents a patient who was suffering from acute disk displacement without a reduction of tenderness on palpation and continuous pain at rest. In ten weeks after the first treatment with PBM using 1064 nm Nd:YAG laser, the clinical image evolved from the acute to chronic phase, and most of the symptoms resolved. In this case, there was no need to prescribe muscle relaxant drugs due to a very good patient's reaction to PBM therapy. The occlusal bite therapy and occlusal reconstruction were not introduced during the acute phase. The improper position of the disk and lack of the remodeling of the retrodiscal tissue can cause the incorrect fixation of the condyle, disk, and fovea [34]. Therefore, it can be hypothesized that in the presented case, the proposed procedure contributed to the significant reduction of time needed for the transition from the acute to the chronic phase (from at least one year to ten weeks).

The use of PBM for TMD is not entirely new. Initial studies exploited handpieces with focal spot surface areas of 0.3–0.6 cm^2^ with Gaussian beam profile delivery [21], meaning that the laser intensity was strongest at the center of the treatment spot and substantially decreased towards the outer edges of the spot. Compared with standard Gaussian-profiled handpieces, flat-top handpieces, such as the one used in this study, are reported to have improved PBM efficacy on differentiation of pre-osteoblastic cells [14]. In this study, we used a 1 cm^2^ surface area handpiece with a flat-top beam profile and collimated beam (Genova®). This type of handpiece is less operator-dependent and can offer improved reproducibility of treatments due to uniform power distribution, especially in clinical situations with larger treatment areas or where the distance from the target may vary during the treatment [22].

## 4. Conclusion

PBM with 1064 nm Nd:YAG laser using a flat-top handpiece seems to be an efficient method for the management of orofacial pain in patients with chronic TMJ disk displacement and may reduce the recovery time of the patient. The method appears comfortable for both the patient and the practitioner, requires only a couple of short (a few minutes long) therapeutic sessions, and is therefore much less subject to the problem of patient compliance. No adverse effects were reported for this clinical case. Appropriate safety eyewear is required for both patients and practitioners. Controlled studies with larger numbers of patients are needed to additionally confirm our results.

## Data Availability

Images included and patient's data are with Dr. Regulski and Dr. Szopinski.
